# Mechanical Compliance and Immunological Compatibility of Fixative-Free Decellularized/Cryopreserved Human Pericardium

**DOI:** 10.1371/journal.pone.0064769

**Published:** 2013-05-21

**Authors:** Maria Cristina Vinci, Giulio Tessitore, Laura Castiglioni, Francesca Prandi, Monica Soncini, Rosaria Santoro, Filippo Consolo, Francesca Colazzo, Barbara Micheli, Luigi Sironi, Gianluca Polvani, Maurizio Pesce

**Affiliations:** 1 Laboratorio di Ingegneria Tissutale Cardiovascolare, Centro Cardiologico Monzino-IRCCS, Milan, Italy; 2 Dipartimento di Scienze Cardiovascolari, Università degli Studi di Milano, Milan, Italy; 3 Dipartimento di Farmacologia, Università di Milano, Milan, Italy; 4 Dipartimento di Bioingegneria, Politecnico di Milano, Milan, Italy; 5 Banca dei Tessuti Cardiovascolari della Regione Lombardia, Milan, Italy; University of Rochester, United States of America

## Abstract

**Background:**

The pericardial tissue is commonly used to produce *bio*-prosthetic cardiac valves and patches in cardiac surgery. The procedures adopted to prepare this tissue consist in treatment with aldehydes, which do not prevent post-graft tissue calcification due to incomplete xeno-antigens removal. The adoption of fixative-free decellularization protocols has been therefore suggested to overcome this limitation. Although promising, the decellularized pericardium has not yet used in clinics, due to the absence of proofs indicating that the decellularization and cryopreservation procedures can effectively preserve the mechanical properties and the immunologic compatibility of the tissue.

**Principal Findings:**

The aim of the present work was to validate a procedure to prepare decellularized/cryopreserved human pericardium which may be implemented into cardiovascular homograft tissue Banks. The method employed to decellularize the tissue completely removed the cells without affecting ECM structure; furthermore, uniaxial tensile loading tests revealed an equivalent resistance of the decellularized tissue to strain, before and after the cryopreservation, in comparison with the fresh tissue. Finally, immunological compatibility, showed a minimized host immune cells invasion and low levels of systemic inflammation, as assessed by tissue transplantation into immune-competent mice.

**Conclusions:**

Our results indicate, for the first time, that fixative-free decellularized pericardium from cadaveric tissue donors can be banked according to Tissue Repository-approved procedures without compromising its mechanical properties and immunological tolerance. This tissue can be therefore treated as a safe homograft for cardiac surgery.

## Introduction

Pericardium is employed in cardiac surgery to repair congenital heart defects or to perform valve reconstruction. In addition, pericardium of animal origin (pig, cow) is the elective material to fabricate bio-prosthetic valves to be employed in surgical replacement of insufficient and/or stenotic valves. Indeed, thanks to its abundant collagen bundles and elastic fibers composition, pericardium has a relatively high resistance to mechanical stress, comparable to that of the native heart valves leaflets.

The technology commonly used to produce heart valves leaflets from animal-derived pericardium consists in treating the tissue with low concentration aldehydes (e.g. glutaraldehyde, GA), necessary to create chemical bonds among the extracellular matrix (ECM) components, thereby further increasing the tissue mechanical resistance, and to prevent acute host immune rejection [1]. On the other hand, aldehyde treatment has also drawbacks regarding the tissue long-term durability. In fact, clinical data from long term follow up of patients receiving pericardium-made bio-prosthetic valves, has clearly indicated a severe impact of structural valve deterioration (SVD) and calcification, due to permanence of fixative remnants having potent cytotoxic effects. In addition, GA fails in removing animal-specific antigens such as the (α1, 3)-Gal epitope [2]–[6], which elicits chronic rejection of the implanted prostheses. Importantly, the impact of SVD is highly correlated to the age of recipients, with younger patients being un-favored - and needing redo strategies - as early as 10–15 years following the first surgery in a significant number of cases [7], [8].

In order to overcome these limitations, basic research has actively pursued fixation-free protocols (i.e., avoiding the use of aldehydes) aimed at enhancing pericardial tissue durability. In particular, novel decellularization methods have been proposed, based on treatment with hypotonic “decellularization” buffers, containing detergents and DNA/RNA digesting enzymes to eliminate cell debris and the remaining nucleic material from the tissue. These strategies have been employed in the production of both animal [9] and human-derived [10], [11] pericardium with relevant implications for clinical use. Moreover, they have been proposed for the generation of tissue engineered heart valves (TEHVs) [12].

Despite a very active research in this field, animal-derived and human-derived decellularized pericardium have not been yet introduced in the routine clinical practice. This also depends on the absence of convincing evidences that this tissue may be prepared and cryopreserved according to Tissue Repository-compliant procedures, without compromising its mechanical properties and immunological tolerance. In the present study, we aimed at filling this technological gap by setting up a fixative-free decellularization process followed by tissue cryopreservation procedure of human pericardial tissue obtained by cadaveric tissue donors. We validated our protocol with specific histological and biomechanical analysis of the tissue, which revealed, *i)* a preserved structure of the collagen bundles and elastin fibers, and *ii)* the maintenance of suitable mechanical properties. Remarkably, the immunological compatibility of decellularized/cryopreserved pericardium was proven by subcutaneous transplantation for up to 60 days into immune-competent mice. From these results, we conclude the feasibility of a Tissue Repository approved procedure for the decellularization and cryopreservation of pericardial samples from tissue donors that may be released as a novel homograft tissue.

## Results

### Maintenance of tissue integrity and complete genetic material removal by fixative-free decellularization procedure

**Figure 1:**
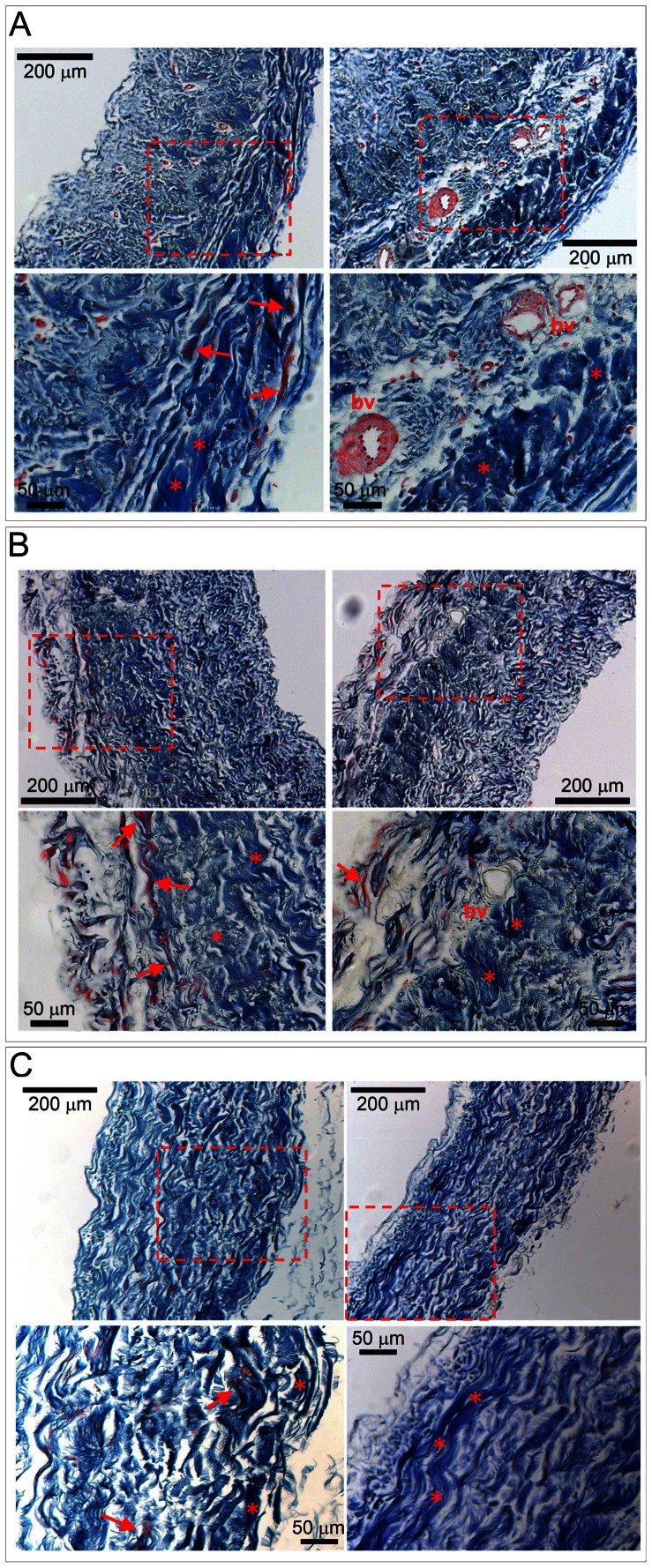
Histological appearance and decellularization of pericardial samples. (A, B, C) Masson's trichrome staining of the pericardial tissue before (A), after decellularization (B) and after decellularization/cryopreservation (C). Upper pictures in each panel show low magnification of the tissue, while lower pictures show magnification of the boxed areas. In these pictures it is evident that decellularization did not affect the structure of collagen bundles (*) and of elastic fibers (arrows). Bv: blood vessels.

**Figure 2:**
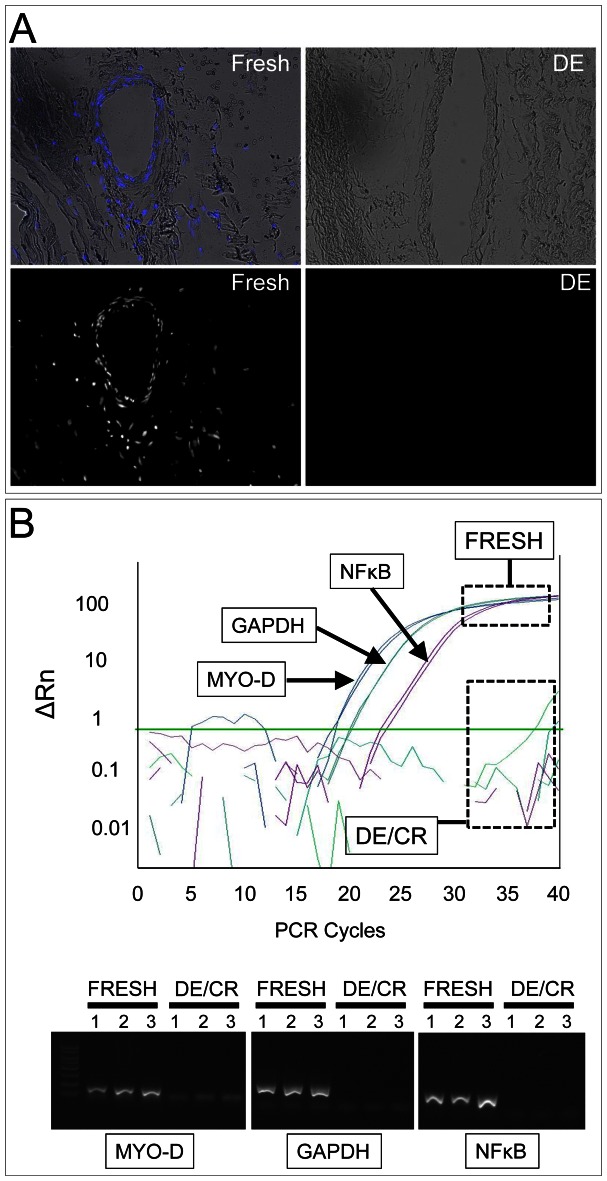
Efficiency of the decellularization process. (A) Hoechst 33258 staining was used to histologically assess the performance of the decellularization process. The upper panels show the overlapped bright field and Hoechst dye channels of the fresh and DE tissues without counterstaining, while lower panels show the dark field image of the Hoechst dye channel only in the same microscopic field. The lack of any signal in DE samples indicates a complete removal of double strand DNA from the tissue. (B) Q-PCR on DNA extracted from fresh and DE/CR samples shows complete removal of DNA by decellularization procedure using primers specific for the *hMYO-D*, *hGAPDH* and *hNFκB* genes promoter/coding sequences. The upper panel shows a representative amplification plot from one of the three FRESH samples with each primers pair, while the curves under the green bar are under-threshold signals generated by the corresponding DE/CR sample. The lower panel shows an agarose gel run of the PCR amplification products; as expected, no amplification bands were obtained by none of the DE/CR samples.

Histological analysis (Fig. 1A, 1B and 1C) was used as a first line of inspection to qualitatively assess, *i)* the efficiency of cells removal after decellularization, *ii)* the degree of the tissue histo-architecture preservation, and *iii)* the content and distribution of the collagen bundles and elastic fibers in the extracellular matrix (ECM). In detail, Masson's staining of decellularized (DE) or decellularized/cryopreserved (DE/CR) pericardium transversal histological sections showed a complete removal of cells. The treatment was not found to cause major deterioration of the tissue, as witnessed by preservation of collagen bundles and elastic fibers. In addition, DE or DE/CR samples did not appear swollen compared with fresh tissue. To verify the removal of cells, staining with Hoechst 33258 of the histological sections was also performed [10], followed by fluorescence microscopy analysis. As shown in Figure 2A, this showed an efficient removal of cells, with no evidence of cellular or nuclear residues observed in the sections. Absence of remaining genetic material from DE/CR samples was confirmed by Real Time-PCR on DNA extracted from three pericardium samples, using primer couples specific for the *hMYO-D*, *hGAPDH* and *hNFκB* genes promoter/coding sequences (Figure 2B, Table 1).

**Table 1:** CT values (mean±SE) obtained by real time PCR amplification of DNA extracted from FRESH and DE/CR samples.

	CT (mean±SE)	
GENE	FRESH	DE/CR
MYO-D	19,25±0,54	0
GAPDH	26,82±2,83	0
NFκB	18,68±0,53	0

Each amplification was performed in duplicate using 3 independent FRESH and DE/CR samples.

### Mechanical integrity of human pericardium after cryopreservation procedure

**Figure 3:**
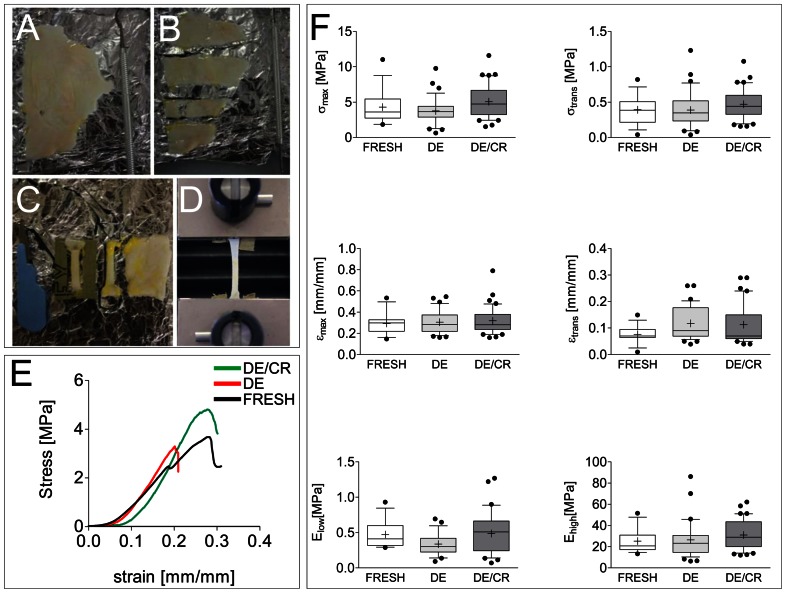
Uniaxial mechanical testing of fresh, DE and DE/CR pericardial samples. (A–D) Preparation steps of pericardial specimens; intact pericardium (A), strips of pericardium (B), dog-shaped specimens obtained from the strips (C), specimen mounted into the clamps of the uniaxial test machine (D). (E) Representative curves showing the stress/strain relationship of fresh, DE and DE/CR samples. (F) Graphical representation of the six mechanical parameters obtained for fresh, DE and DE/CR samples: elastic modulus at low (E_low_) and high (E_high_) strain values, transition stress (σ_trans_) and strain (ε_trans_), maximum tensile stress (σ_max_) and strain (ε_max_). Results are graphically represented with inter-quartile range box plots and whiskers indicating 10^th^ and 90^th^ percentile, plus outlier values (•). Continuous lines in box plots indicate median values of each data set, while crosses indicate mean of the same data. None of these values in DE or DE/CR samples were significantly different from fresh tissue values as verified by Kruskall-Wallis test (n = 15, fresh samples; n = 36, DE samples; n = 41, DE/CR samples; pericardial tissues from 3 independent cadaveric donors).

Uniaxial tensile loading (UTL) tests were carried out on fresh, DE and N_2_ vapors-cryopreserved DE pericardium (DE/CR) samples to quantitatively characterize the biomechanical properties of the tissue. In particular, we used UTL tests to reveal any potential alteration of the biomechanical characteristics of the tissue induced by the cryopreservation procedure. Figure 3A–D shows the preparation steps of pericardial specimens for UTL tests, while 3E represents three typical stress/strain curves obtained from fresh, DE and DE/CR specimens strained to rupture. The stress-strain behavior for each specimen of the three groups was analyzed by means of six parameters [13], including: the elastic modulus (i.e., the stress-strain curve slope) at low (E_low_) and high strain (E_high_), representative of the tissue resistance due to the contribution of the elastin and collagen fibers composing the ECM, respectively; the tensile stress (σ_trans_) and strain (ε_trans_) values at the transition between the elastin and collagen stress-strain curve slope; the maximum tensile stress (σ_max_) and strain (ε_max_) characterizing the failure phase of the tissue sample. The comparison of the six parameters in fresh *vs.* DE and DE/CR groups did not show statistically significant differences (Fig. 3F), suggesting that the procedure adopted to obtain the decellularized pericardium not modify the tissue resistance to mechanical strain. This result suggests that the integrity and the arrangement of ECM components were maintained in DE and DE/CR pericardium samples, ensuring a biomechanical performance similar to that of native tissue.

### Immunological compatibility of decellularized pericardium, before and after cryopreservation

**Figure 4:**
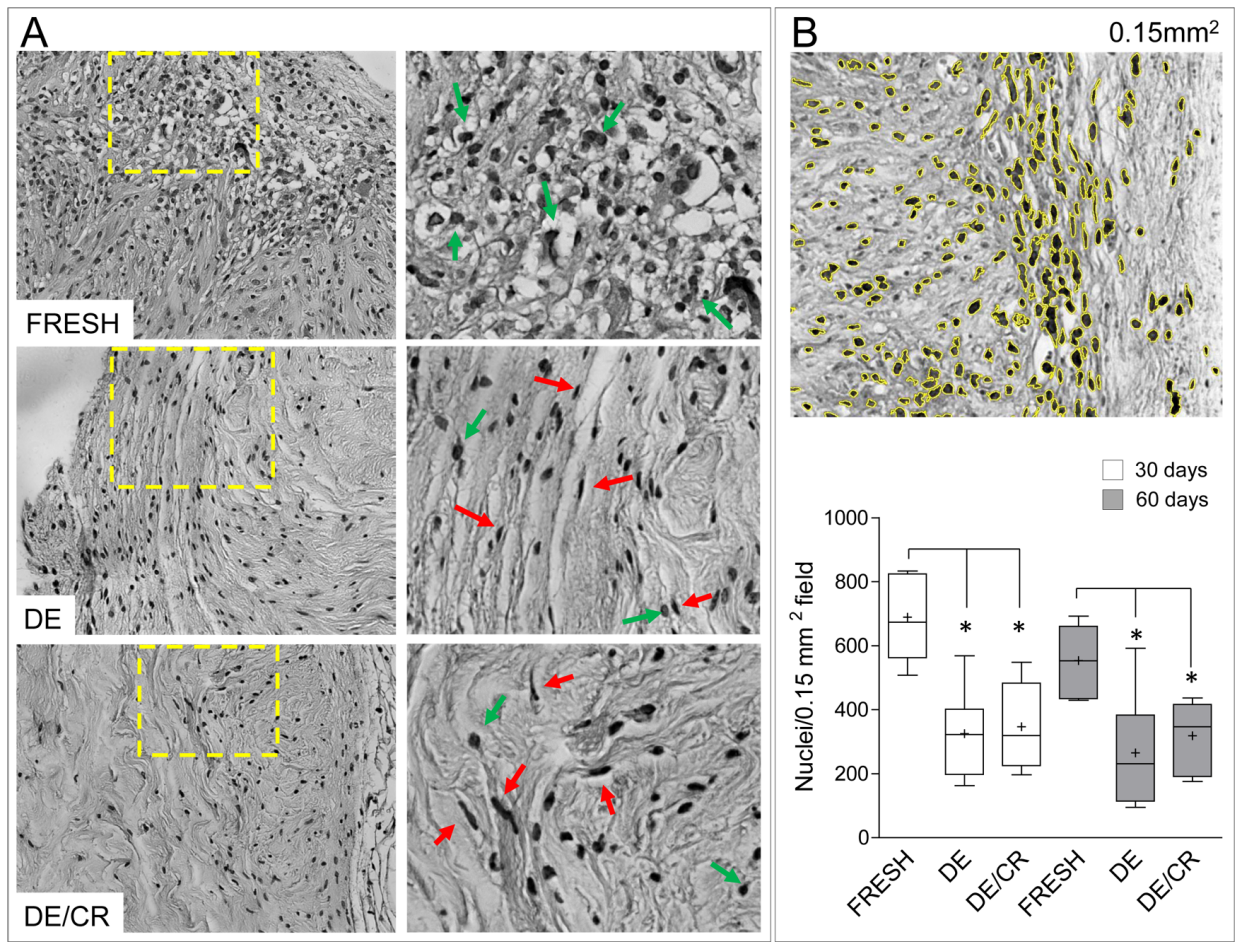
Gross morphology and host cell invasion quantitative analysis at 30 and 60 days following fresh, DE and DE/CR tissues implantation into imunocompetent mice. (A) Left panels show low magnification of fresh, DE and DE/CR pericardium after recovery from the subcutaneous position at 60 days. It is evident an overall higher number of cells in fresh *vs.* DE and DE/CR samples. Foci of inflammation with signs of tissue absorption were often observed in fresh samples, while these were never found in both DE and DE/CR tissues, indicating lower rejection. Panels on the right show high magnification of the yellow-boxed areas in left panels. It is evident that in fresh samples, infiltration was mainly due to cells with round nuclei, resembling inflammatory cells (green arrows), while the majority of infiltrating cells in DE and DE/CR pericardium specimens had elongated nuclei, resembling fibroblasts (red arrows). (B) Quantification of infiltrating cells was performed by computer assisted nuclei counting after appropriate filtering of the B/W images. Picture at the top is a 0.15 mm^2^ micrograph where nuclei were automatically recognized and contoured in yellow by Image-J software; box-plot in the bottom indicates the results of nuclei counting at 30 and 60 days in fresh, DE and DE/CR samples. Results are graphically represented with inter-quartile range box plots and whiskers indicating 10^th^ and 90^th^ percentile. Continuous lines in box plots indicate median values of each data set, while crosses indicate mean of the same data. * indicates *P*<0.05 for statistical comparison of fresh *vs.* DE and DE/CR samples at both time points by Kruskall-Wallis test with Dunn's post-*hoc* analysis (5≤n≤8).

The immunological compatibility of human- and animal-derived pericardium has been previously tested by transplantation in animals, where the reaction of the host against the graft, and the graft calcification were assessed mainly by *ex-post* histological analysis of inflammatory/immune cells invasion [11], [14]–[16]. To assess whether the decellularization and the decellularization/cryopreservation procedure of the human pericardium adopted in the present study led to changes in the tissue immune-tolerance, we adopted a similar strategy. Fresh, DE and DE/CR pericardium fragments were transplanted in subcutaneous position into immune-competent mice for 30 and 60 days followed by histology, immunohistochemistry and analysis of inflammatory-competent cells in peripheral circulation. As shown in Figure 4A, the histological appearance of DE and DE/CR samples was strikingly different from that of the fresh tissue. In fact, high levels of round cells with macrophage/lymphocyte morphology, forming deep layers of granulation tissue were observed in fresh pericardium samples; by contrast an overall lower number of cells with a predominant spindle-shape morphology and elongated nuclei, was observed in in DE and DE/CR samples. Of note, foci of donor-derived inflammatory cells infiltration and tissue matrix reabsorption were never observed in any of the DE and DE/CR samples explanted from mice at both time points, indicating a high - but not different - degree of compatibility of DE and DE/CR *vs.* fresh pericardium. To confirm the overall lower infiltration by host cells, an automatic counting of cellular nuclei was performed in fixed area images (0.15 µm^2^) of hematoxyilin/eosin stained sections of fresh, DE and DE/CR samples. Results of this survey confirmed an overall lower presence of cells in DE and DE/CR compared with fresh pericardial samples (Fig. 4B).

We and others have found that patients receiving allograft valve transplantation show increased serum levels of anti HLA-1 antibodies [17] and of circulating cytotoxic and helper T-lymphocytes (CTLs, HTLs) [18], [19].

**Figure 5:**
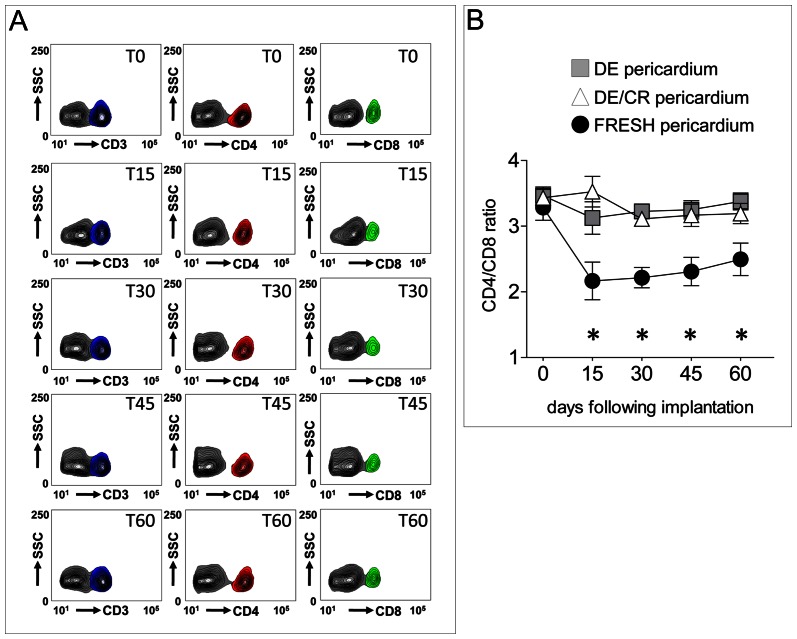
Dynamics of CD3^+^, CD4^+^ and CD8^+^ cells mobilization in mice implanted with fresh, DE and DE/CR samples. (A) Representative flow cytometry detection of CD3^+^ (left contour plots column), CD4^+^ (center contour plots column) and CD8^+^ (right contour plots column) circulating cells in a mouse receiving a non decellularized (FRESH) pericardial sample at 0, 15, 30, 45 and 60 days. CD3^+^, CD4^+^ and CD8^+^ cells are indicated, respectively by blue, red and green contours on the right of the main PBMNCs population (grey contours), established by staining with isotype control antibodies. (B) CD4^+^/CD8^+^ lymphocytes ratio was determined at all the time points and plotted. Mice receiving fresh pericardium showed a reduced ratio suggesting increase in the number of circulating T-lymphocytes implicated in T cell-mediated tissue rejection. * indicate a statistical difference (*P*<0.05, by two-ways ANOVA with Bonferroni post-*hoc* test, n = 5) between the CD4/CD8 ratio in mice receiving fresh *vs.* those implanted with DE and DE/CR tissues.

Since an elevation of inflammatory markers and immune cell circulation has been indicated as one of the main causes of SVD, a time course analysis of circulating T-lymphocytes was performed in blood samples obtained from mice receiving fresh, DE and DE/CR pericardium at 15, 30, 45 and 60 days after implantation. As a reference for the dynamics of the T-cell mediated rejection of pericardial tissue, the relative ratio of the CD4^+^/CD8^+^ lymphocytes was calculated after appropriate recognition of total circulating T-lymphocytes stained with the *pan* T-cell marker CD3. As shown in Figure 5, a statistically significant decrease of this ratio, caused by a relative increase of circulating CD8^+^ cells, was observed at all times in mice receiving fresh pericardial samples. By contrast, a constant CD4^+^/CD8^+^ cell ratio around or above 3 was observed in mice implanted with DE or DE/CR, suggesting absence of a strong T-cell-mediated immune response. Finally, no statistically significant difference between DE and DE/CR receiving mice was observed, suggesting that cryopreservation did not alter the immunological compatibility of human decellularized pericardium.

**Figure 6:**
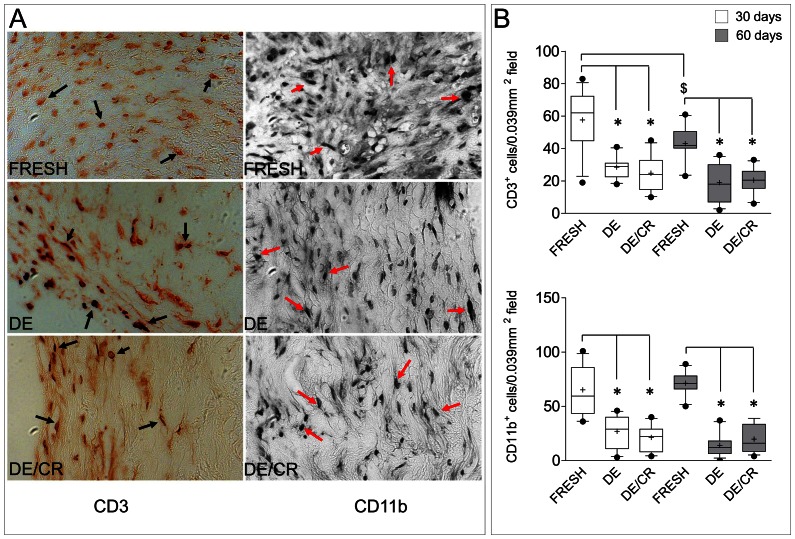
Infiltration of the implanted pericardial tissue by CD3^+^ lymphocytes and CD11b^+^ macrophages. (A) Representative images of fresh, DE and DE/CR pericardium tissue sections recovered by a mouse at 60 days and stained with CD3− and CD11b-specific antibodies. Black arrows and red arrows indicate, respectively CD3^+^ and CD11b^+^ cells present in the tissue. Note the higher number of cells in fresh pericardial specimens compared to DE or DE/CR samples. (B) Quantification of CD3^+^ (upper plot) and CD11b^+^ cells (lower plot) by software-assisted manual counting (Image J) into fixed (0.039 mm^2^) tissue sections areas. At both time points the number of CD3^+^ and CD11b^+^ cells was significantly higher in mice receiving fresh vs. DE or DE/CR pericardium (* indicate *P*<0.05 by 1-way ANOVA with Newman-Keuls post-*hoc*; n = 12). Interestingly, the number of lymphocytes was significantly lower at 60 days compared with 30 days in mice receiving fresh pericardium ($ indicates P<0.05 by Mann-Whitney rank sum test; n = 12).

The host immune response has a striking impact on *in vivo* durability of GA-fixed and fixative-free *bio*-prosthetic valve grafts, due to chronic inflammatory process [20] and calcification [21], which leads to progressive SVD and decay of mechanical performance. To reveal the inflammatory cellular species invading the implanted pericardium specimens, an immunohistochemistry staining of fresh, DE and DE/CR samples was performed using anti mouse CD3 and CD11b antibodies, which recognized host-derived lymphocytes and macrophages, respectively (Fig. 6A). The results showed the presence of an elevated number of CD3^+^ and CD11b^+^ cells in fresh pericardial samples explanted at 30 and 60 days; these numbers were significantly higher than in DE or DE/CR explanted pericardial tissue (Fig. 6B). Interestingly, a lower T cells number in fresh pericardium was observed at 60 compared with 30 days, suggesting an attenuation of the host T-cell-mediated immune rejection at longer times post-implantation. A similar decrease in macrophages (CD11b^+^ cells) content was not observed, showing a sustained innate immunity response.

Finally, to assess whether calcification secondary to inflammatory/immune response occurred in the transplanted pericardial specimens, Von Kossa staining was performed on histologic sections of tissue explants at 60 days post-surgery. As shown in Figure S1, no staining was observed in DE or DE/CR explanted samples; small calcium deposits were occasionally found in fresh pericardium specimens recovered after 60 days, showing that inflammatory response was not associated to major pericardium calcification in any of the conditions considered in the present study.

## Discussion

Devising novel procedures to produce replacement allograft or bio-prosthetic valve conduits offers significant expectations to ameliorate the clinical outcome of the current heart valves disease treatments. In fact, the durability of these devices is still unacceptably limited, with an elevated burden for the patients' quality of life. As an example, studies reporting the clinical outcome of patients receiving bio-prosthetic valve implants clearly indicated a progressive SVD due to host inflammatory response and immune rejection. Strikingly, the clinical data indicated a higher propensity of younger subjects susceptible to develop implant failure more rapidly than older patients and thus requiring re-operation [7], [8]. Depending on the type of the employed prostheses, different causes are at the basis of the insufficient durability. For example, the employment of non-decellularized/cryopreserved valve homografts obtained from cadaveric tissue donors does not resolve the problem of the allogenic (man to man) immune response [20], while the employment of the so called “biological” valves - valve conduits of animal origin (porcine valves) or bio-prosthetic valves fabricated with leaflet mimicking biological tissues (typically pig and cow pericardium) - has its major limitation in the use of GA-based fixation protocols, which leave residual free aldehyde groups in collagen abundant tissues [22], form chemical bonds between ECM components, and fail in removing xeno-antigens such as the (α1, 3)-Gal epitope [5], [6], recognized as “non-self” by the human species. Although advanced protocols to reduce the overall cytotoxicity of GA-fixed tissues have been devised [14], [16], [23]–[25], novel procedures to prepare leaflet and pericardial tissue patches preventing the use of aldehydes have been also explored. These procedures are based on decellularization of the tissue through osmotic lysis followed by exposure to ionic (e.g. SDS) and/or non-ionic (e.g. Triton-X 100) detergents to efficiently remove cell remnants. The apparent advantage of these procedures over the GA-based fixation is the lack of aldehyde residues, the absence of chemical bonds between ECM components, and the removal of xeno-antigens. On the other hand, whether decellularized tissues have or not an improved immunologic compatibility and an optimal mechanical performance is still matter of debate requiring further investigation [4], [9], [26].

To our knowledge, human-derived pericardium decellularized with an osmotic/detergent-based procedure has not yet clinically employed. In fact, preservation of mechanical properties and *in vivo* compatibility of this tissue were previously assessed [10], [11]; however, the absence of data related to the maintenance of the tissue material properties after a cryopreservation storage period does not allow conclude whether this tissue may be eventually routinely prepared, banked and distributed by officially recognized homograft Repositories. This particular issue is not trivial. In fact, similar to the production process of cells for cell-based therapy according to the Good Manufacturing Practice [27], the homografts approval for clinical employment requires crucial validation steps to demonstrate the biological and the functional integrity of the tissue. In this regard, the UTL tests performed in the present study (Figure 3) showed that none of the parameters describing the mechanical properties of the human pericardium were modified by the decellularization or the decellularization/cryopreservation procedure. Thus, our data extend the observations by Mirsadraee et al. [10] and underline the relevance of fixation-free decellularization procedures to prepare human pericardium with intact mechanical properties for future clinical employments.

The result of subcutaneous implantation into immune-competent mice was performed taking as a reference the human fresh pericardium. This was done to assess the efficiency of the decellularization protocol at removing cellular material representing a source of allogenic tissue rejection, or promoting secondary calcification. As shown in Figure 4, the overall invasion by host cells was significantly lower in mice receiving DE and DE/CR tissue samples; moreover, in both cases, the number of infiltrating cells was significantly lower than in mice implanted with fresh pericardium. In addition, DE and DE/CR samples showed no signs of reabsorption and formation of granulomas. This finding is in line with the lower numbers of CD3^+^ cells and CD11b^+^ cells in DE and DE/CR compared with the fresh pericardium (Fig. 6) [11]. To further explore the role of systemic inflammation in mice implanted with human pericardial samples, the ratio of circulating CD4^+^/CD8^+^ lymphocytes, a parameter that is commonly taken as a reference to monitor rejection in solid organ transplantation [28] and mouse models [29], was also determined. These tests (Fig. 5) revealed a striking increase in the relative amount of circulating CD8^+^ cells as early as at 15 days post-implantation in mice receiving the fresh pericardium, suggesting a rapid initiation of the rejection process. By contrast, DE and DE/CR pericardial implants were never found to cause an imbalance in lymphocytes ratio for the whole duration of the experiment. Although we did not specifically investigate the subclasses of CD8^+^ circulating cells, this observation appears particularly relevant in the view of homograft transplantation of the DE/CR tissue. In fact, GA-fixed pericardial implants are known to cause an increase of circulating cytotoxic [18] or helper [19] T cells and production of anti HLA-I antibodies, which correlate with graft failure [17]. Thus, the fixative-free decellularization/cryopreservation method used in the present study may be helpful to reduce the inflammatory and cell-mediated immune response in recipients, improving the long-term durability of the implants.

In summary, thanks to unaltered mechanical properties, the potentially reduced cytoxicity and the lowered immunogenicity, we propose the decellularized human pericardium produced with the protocol described here as a novel and safe homograft tissue to be clinically employed. In addition, since the adoption of fixative-free decellularization strategies is recommendable for production of biological-derived material for recellularization with living cells and derivation of TEHVs [16], [25], [30]–[33], the procedure described here may also be useful for devising novel living implants, with higher degree of immunological compatibility and lower potential for SVD, with significant benefits for the patients.

## Materials and Methods

### Ethics statement

Human pericardial samples were obtained as discharge material during heart dissection in the routine processing of valve homografts preparation at the Lombardy Cardiovascular Tissue Repository, the official Lombardy's Facility for collection of cadaveric cardiovascular tissues and cardiovascular homograft preparation and storage (www.cardiologicomonzino.it/Clinica/ChirurgiaCardiovascolare); authorization number: DGR VII/12848 (April 28, 2003 – Official Lombardy Bullettin). Samples from a total of six cadaveric tissue donors were used in the present study. Collection of hearts from these donors was performed after signature of an approved informed consent by donor relatives.

The procedures concerning animal care, surgery, and euthanasia were carried out in accordance to the “Guide for the Care and Use of Laboratory Animals” and the Helsinki declaration. They were further compliant with European directives and guidelines (Legislative Decree September 19, 1994, n. 626; 89/391/CEE, 89/654/CEE, 89/655/CEE, 89/656/CEE, 90/269/CEE, 90/270/CEE, 90/394/CEE, 90/679/CEE). The procedures used in the present study, were further approved and authorized by the Italian Ministry of Health and the University of Milan Ethical Committee (approval number:1242003-A 13/10/2003).

### Decellularization procedure and histology

In the present study, a decellularization procedure similar to that developed by Mirsadree et al. [10], [11] was adopted. After an accurate surgical elimination of fat, pericardial tissue was first washed for 90 min in phosphate-buffered saline (PBS) containing protease inhibitors (aprotinin, 10 KIU/ml, Trasylol, Bayer, Germany; 0.1% w/v EDTA, BDH, Lutterworth, United Kingdom) under continuous agitation, and with buffer changing every 30 min. To achieve cells removal from the tissue, the tissue was treated with hypotonic buffer (10 mM Tris-HCl; pH 8.0) for 16 h under continuous agitation at 4°C in the presence of protease inhibitors, followed by incubation for 24 h in 0.1% (w/v) sodium dodecylsulphate (SDS) in hypotonic buffer at room temperature under continuous agitation. To remove nucleic acid material, the samples were washed in sterile PBS three times for 30 min under agitation and incubated for 3 h at 37°C in a reaction buffer containing 50 U/mL deoxyribonuclease I from bovine pancreas [DNase, Sigma- Aldrich, Germany] and 1 U/mL ribonuclease A from bovine pancreas [RNase, USB] in 10 mM Tris-HCl (pH 7.5; Sigma-Aldrich) under gentle agitation on a gyrorocker. Finally, samples were washed in PBS for three periods of 30 min under agitation. To check the completeness of cell removal, pericardial tissue was fixed immediately after the end of the decellularization procedure and embedded into paraffin. Tissue sections (4–6 µm) were cut and stained with ematoxylin/eosin, Masson's trichrome solution, Von Kossa staining solution, or with Hoechst 333258 nuclear dye.

### DNA extraction and Q-PCR methods

Total DNA was isolated with TriPure Isolation Reagent (Roche Applied Science) from 100 mg of fresh and DE pericardium samples. Gycogen was added to samples before precipitation to see pellets. Quantitative real-time PCR (qRT-PCR) amplifications were carried out for MYOD, GAPDH and NFkB gene promoters using Power SYBR Green PCR Master Mix (Applied Biosystems) on a 7900 Fast Real-Time PCR System (Applied Biosystems). Primer sequences were as follows:

**hMYOD promoter:** sense-5′-CCTCTTTCGGTCCCTCTTTC-3′, antisense-5′-ATGGGTAGAGCGGCTGTAGA-3′;

**hGAPDH:** sense-5′-TACTAGCGGTTTTACGGGCG-3′, antisense-5′ –TCGAACAGGAGGAGCAGAGAGCGA-3′;

**hNFκB promoter:** sense-5′-CAGCCGATGAGAGCCGGCAG-3′, antisense-5′-CCGGACCTCCCAGCCTGACA-3′.

### Cryopreservation and thawing

Pericardium samples were frozen and thawed according to the procedures used by Lombardia Cardiovascular Tissue Bank. Briefly, samples were frozen in a double sterile bag containing freezing solution (RPMI 1640 plus 10% of DMSO) by the use of liquid nitrogen ultra-low-temperature cooler (Planer - SOL S.p.A., Milan, Italy) and stored in N_2_ vapors in temperature range between −140°C and −185°C. The samples were thawed inside the sterile bag and washed three times in fresh RPMI 1640 [34], [35] immediately before use.

### Mechanical testing

Uniaxial tensile loading (UTL) was performed on native (fresh), decellularized (DE) and decellularized/cryo-preserved (DE/CR) pericardial samples. Before the test, the samples were maintained immersed in saline solution at 4°C. Dog-bone shaped specimens of 4 mm width and 20 mm height were dissected; in detail, 15, 36 and 41 specimens were retrieved and tested from the fresh, DE and the DE/CR groups, respectively. No preferential orientations were considered when cutting the specimens, assuming pericardium isotropy [10], [36]. The thickness of each specimen was measured by means of a micrometer (0.05 mm of scale) in 3 different positions along the specimen height and the mean value (*t*_av_) was used for mechanical parameters calculation. The specimens were then mounted onto the clamps of the testing machine; a sandpaper frame was constructed around the specimen to facilitate uniform gripping and to avoid sample slippage [37]. Tissue specimens were preloaded up to 0.01 N and subjected to a number of preconditioning loading-unloading cycles (ranging from 6 to 8 cycles), at 15% maximum strain with an elongation rate of 10 mm/min, until the loading-unloading curves were almost superimposed. After tissue preconditioning the specimen was preloaded up to 0.01N, the specimen initial length (*l*_0_) was measured, and UTL test conducted until specimen failure, at a constant velocity of 10 mm/min. The samples were maintained immersed in PBS at 4°C until the UTL tests begun. All the tests were performed at room temperature and the specimens were maintained hydrated with PBS for the whole test duration.

The load (*F*) and displacement (*l*) data were acquired from the load cell and the stroke of the cross-head of the testing machine and the engineering stress (σ) and strain (ε) were calculated for each data point, according to equation 1 and 2:
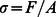
(1)

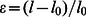
(2)where *A* is the cross-sectional area of the specimen at zero strain, expressed as (eq. 3):
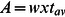
(3)with, *w*, *t_av_* and *l*_0_ representing the specimen width, average thickness, and length at zero strain, respectively.

The stress strain behavior was described by means of 6 parameters according to Korossis et al. [13]: elastic modulus at low (E_low_) and high (E_high_) strain values, transition stress (σ_trans_) and strain (ε_trans_), maximum tensile stress (σ_max_) and strain (ε_max_). The biomechanical parameters calculated for verifying pericardium mechanical properties are based on engineering stress and strain values assuming that the cross-sectional area reduction is negligible during specimen deformation. Even if the real calculated stress, considering the cross-sectional reduction of the specimens may be more accurate, its measurement requires the use of optical-based systems, equipment not commonly integrated in the standard UTL apparatuses. On the other hand, most of the published UTL data consider the calculation of engineering stress and strain [10], [35], [36], [37].

### Surgical procedures, peripheral blood collection, pericardium recovery and image analysis

Subcutaneous implantation of pericardium fragments (10×10 mm) was performed in male CD1 mice (n = 54; 9 mice/group) (Charles River, Calco Italy) of about 30gr weight. Before implantation, mice were anesthetized with a ketamine (100 mg/Kg) - xylazine (20 mg/kg) solution injected intraperitoneally; at the same time, blood sampling was performed. Peripheral blood was also collected in 5% EDTA containing tubes at 15, 30, 45 and 60 days after implantation following mild anesthesia with isoflurane (1% in 100% oxygen, v/v). Pericardial tissue specimens recovered at 30 and 60 days after implantation were placed into 4% buffered formalin immediately after recovery from the animals and processed for conventional histological analysis (see before) and immunohistochemistry. Tissue sections were observed under light/fluorescence microscopes (Axiovert/Akioskop) equipped with image analysis software (Zeiss, Cell Quest). Images were acquired at a fixed magnification and analyzed using Image-J software (http://rsbweb.nih.gov/ij), after appropriate grayscale filtering, for automatic/manual nuclei/cells counting.

### Immunohistochemistry

For immunohistochemistry, dewaxed and re-hydrated sections (5 µm thick) were incubated with 5% bovine serum albumin (BSA) in PBS to block nonspecific binding. The sections were then immunostained with rabbit mAbs recognizing mouse CD3 (Abcam, Cambridge, UK, ab5690) or CD11b (Abcam, Cambridge, UK, ab75476), followed by biotinylated goat anti-rabbit IgG, using an avidin–horseradish peroxidase–biotin complex method (Vector Laboratories, Burlingam, CA) and DAB or NovaRed substrate (Vector Laboratories). A manual counting protocol of CD3^+^ and CD11b^+^ cells was performed by using Image-J software.

### Flow cytometric evaluation of circulating monocytes

Before staining with antibodies, blood was treated with ammonium chloride to lyse red blood cells. Lymphocyte population (CD3^+^ cells) and CD8^+^ cells were identified by staining with allophycocyanin-conjugated anti-mouse CD3 (Ab553066), and fluorescein-isothiocyanate-conjugated anti-mouse CD8 (Ab553031), while the percentage of CD4^+^ population was evaluated by the use of phycoerythrin-conjugated anti-mouse CD4 antibody (Ab553653). All antibodies were from BD Biosciences, Milan, Italy. Cells were analyzed using a FACSCalibur flow cytometer (BD Biosciences, Milan, Italy). Data on CD4^+^/CD8^+^ cells ratio were derived by acquisition of at least 10^4^ events with appropriate logical gating of the CD3^+^ cellular population.

### Statistical analyses

Statistical comparisons were performed with Graph-Pad 5 (Prism) statistical software. All data were analyzed with Shapiro-Wilk normality test. In case of normal data distribution, statistical comparisons were performed using parametric tests - 1-way/2ways ANOVA with Newman-Keuls/Bonferroni post-hoc analysis. In case of non-normal data distribution, the statistical methods used to compare data were the Kruskall-Wallis test with Dunn's multiple comparison post-hoc analysis or the Mann-Whitney rank sum test. The methods adopted to perform statistical comparisons are indicated in each figure legend.

## Supporting Information

Figure S1 **Von Kossa staining of fresh, DE and DE/CR pericardium specimens recovered from mice at 60 days following implantation.** Except for few small calcium deposits (arrows) in fresh samples, calcification was not observed.
(TIF)
