# Supplementary figures and images for: Mechanical Compliance and Immunological Compatibility of Fixative-Free Decellularized/Cryopreserved Human Pericardium
Source: PLoS One. 2013 May 21;8(5):e64769. doi: 10.1371/journal.pone.0064769 (PMC3660606; doi:10.1371/journal.pone.0064769)

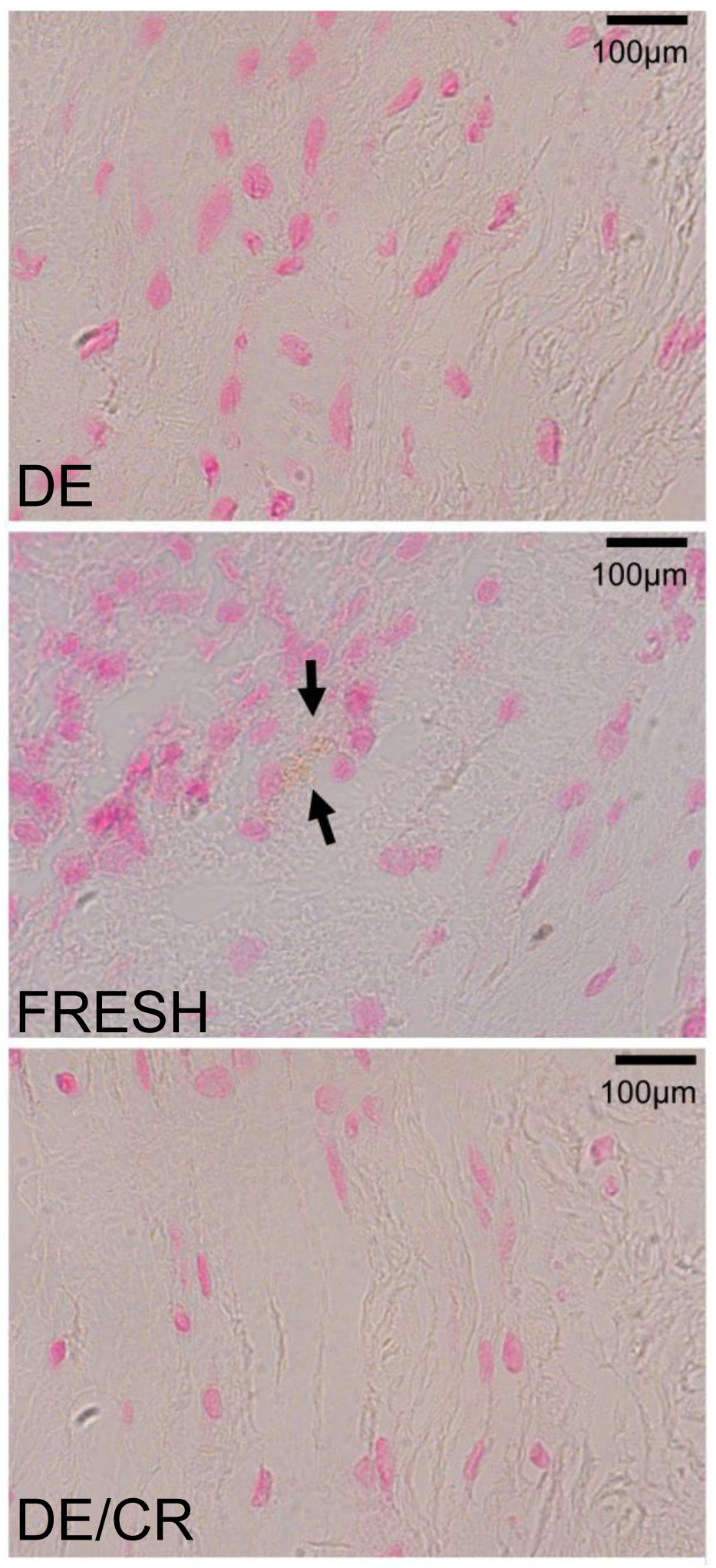

Supplement: Figure S1 — Von Kossa staining of fresh, DE and DE/CR pericardium specimens recovered from mice at 60 days following implantation. Except for few small calcium deposits (arrows) in fresh samples, calcification was not observed. (TIF) [file pone.0064769.s001.tif]
